# Carbon Nanofibers‐Based Anodes for Potassium‐Ion Battery

**DOI:** 10.1002/open.202300286

**Published:** 2024-01-10

**Authors:** Chao Li, Wen‐jun Jiang, Zhen‐yu Liu

**Affiliations:** ^1^ Sinopec Maoming Research Institute 525000 Maoming China; ^2^ Beijing University of Chemical Technology 100000 Beijing China

**Keywords:** energy storage technologies, lithium-ion batteries, potassium-ion batteries, carbon nanofibers

## Abstract

In recent years, with the global warming getting worse and increasing demand for energy, countries around the world are trying to develop new energy storage technologies to solve this problem. Currently, potassium‐ion batteries (PIBs) have attracted tremendous attention from researchers as low‐cost and high‐performance energy storage devices. However, due to the huge ionic radius of K^+^, PIBs face significant volume expansion during cycling, which can easily lead to the collapse of electrode structures. In addition, the poor diffusion kinetics of K^+^ seriously affect the electrochemical performance of the battery. Carbon nanofibers (CNFs)‐based materials (including CNFs, metal/CNFs composites, chalcogenide/CNFs composites, and other CNFs‐based materials) are widely used as PIBs electrode anode materials due to their three‐dimensional conductive network, heteroatom doping and excellent mechanical properties. This review discusses in detail the research progress of CNFs‐based materials in PIBs, including material preparation, structural design, and performance optimization. On this basis, this article explores the key issues faced by CNFs‐based materials and future development directions, and proposes improvement suggestions for providing new ideas for the development of CNFs‐based materials.

## Graphical abstract

This article explores the key issues faced by carbon nanofibers (CNFs)‐based materials and future development directions, and proposes improvement suggestions for providing new ideas for the development of CNFs‐based materials.

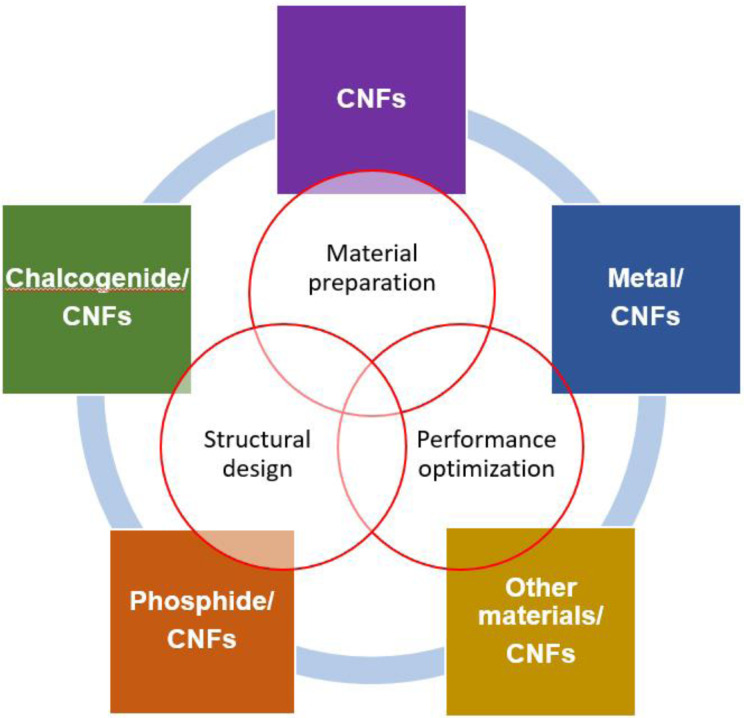

## 1 Introduction

### 1.1 Overview on PIBs

Since entering the 21st century, with the rapid development of mobile devices and electric vehicle industries, energy storage devices have entered a period of rapid development.[^1^, ^2^] Owing to its reusability and high energy density, lithium‐ion batteries (LIBs) have been widely used in the current market. However, with the continuous increase in market demand, the raw material prices and production costs of LIBs remain high, seriously affecting their future development.^[3]^ Therefore, there is an urgent need to develop other high‐performance and cheap energy storage devices.[^4^, ^5^] In recent years, potassium‐ion batteries (PIBs) have attracted widespread research interest due to their abundant raw materials, low cost, and low redox potential (−2.93 V *vs*. standard hydrogen electrode) for K/K^+^. Although PIBs occupy some unique advantages, the ionic radius of K^+^ (1.38 Å) is larger than that of Li^+^ (0.76 Å), which can cause significant volume expansion and contraction for the cell upon cycling, damaging the internal structure of the electrode material and causing irreversible capacity loss.[^6^, ^7^] In addition, the poor diffusion kinetics of K^+^ seriously hinder the capacity and rate performances of the battery. Thus, in order to improve the electrochemical performances of PIBs, electrode materials need to be carefully designed and optimized.

### 1.2 Development of PIBs Anode Material

As one of the key components of PIBs, anode electrode materials greatly affect the electrochemical performance and safety performance of the battery. It is worth mentioning that commercial graphite in LIBs generally undergoes three stages [C→KC_24_ (stage I)→KC_16_ (stage II)→KC_8_ (stage III)] during the intercalation of K^+^, exhibiting high theoretical capacity (279 mAh g^−1^). However, the large volume expansion (61 %) of graphite during cycling leads to the pulverization of the electrodes and the formation of large quantities of the solid electrolyte interphase (SEI) layer on the electrode surfaces, which eventually cause the continuous decline of the capacity. Therefore, promoting the development of PIBs requires continuous exploration of other high‐performance electrode materials. At present, the reported anode electrode materials for PIBs mainly include carbon materials [*e. g*. grapheme, soft carbon, hard carbon and carbon nanofibers (CNFs)], metals (*e. g*. Sn and Sb), metal sulfides (*e. g*.SnS_2_ and CoS_2_), and other materials (*e. g*. Fe_2_P, MoP and MXene‐based materials).[^5^, ^8^, ^9^, ^10^] Among them, CNFs have a special research interest on researchers. Table 1 summaries the electrical conductivity, microstructure, structural advantages, and raw material of CNFs. On the basis of these basic information, CNFs can be considered as an efficient anode for PIBs.[^11^, ^12^, ^13^, ^14^] However, individual CNFs typically exhibit limited discharge capacity in PIBs. Therefore, CNFs typically appear together with other high‐performance materials as anode electrode materials for PIBs.

**Table 1:** Comparison of the electrical conductivity, microstructure, structural advantages, and raw material of CNF synthesis methods.

Synthetic routes	Electrical conductivity (S cm^−1^)	Microstructure	Structural advantages	Raw material
Electrosp‐inning	1–600	Diversified structure (random, core‐shell, and aligned); long and continuous CNFs; the diameter 10 nm to 10 μm	Structures modified by tuning the precursor solutions and electrospinning parameters; porous CNFs	Polyacrylonitrile (PAN), polyvinyl alcohol, polyvinyl pyrrolidone (PVP), polyvinylidene fluoride and pitch
Chemical vapor deposition	1×10^3^ to 1×10^4^	Diversified structure (cup stacked CNFs and the platelet‐stacked CNFs); high aspect ratio; the diameter <1 μm	Formation of CNFs with high quality, and uniform shape	Methane, ethylene, acetylene and propylene
Template‐based strategies	1–10	Porous, tubular, and core‐shell CNFs; the diameter 50 nm to 200 nm	Structure is based on the pore and size of templates	Hard‐templates (*e. g*. aluminum oxide membranes( and solution‐based template (*e. g*. insoluble complex)
Biomass methods	0.2–2	Porous and hollow CNFs; the diameter 10 nm to 80 nm	Highly interconnected with large numbers of junctions; high absorption capacity	Organic polymer nanofibers (*e. g*. bacterial cellulose)

### 1.3 Advantages and Challenges of CNFs‐based Anodes for PIBs

In detail, CNFs‐based anodes for PIBs own the following merits: (i) nanostructures can shorten the diffusion distance of K^+^ and increase the contact area with K^+^; (ii) three‐dimensional (3D) cross‐linked heterogeneous conductive network could inhibit the aggregation of nanoparticles, enhance the electronic conductivity, and thus facilitate the electron/ion transport; and (iii) self‐supporting flexible electrodes simplify the manufacturing process of batteries, and increase the volume energy density of the battery by reducing the use of metal current collectors, binders and conductive additives.[^3^, ^5^, ^15^]

However, CNFs‐based materials (including CNFs, metal/CNFs, chalcogenide/CNFs, phosphide/CNFs and other CNFs‐based materials) still face many challenges such as low first cycle coulombic efficiency, unclear reaction mechanism, complex synthesis method and high cost, *etc*. In this review, the research progress on CNFs‐based materials were summarized. In addition, this work provides unique insights into the experimental methods, structural design, or performances of almost every typical case, which is believed to stimulate the further development for CNFs‐based materials.

## 2 Application of CNFs‐based Materials in PIBs

### 2.1 Application of CNFs in PIBs

**Figure 1:**
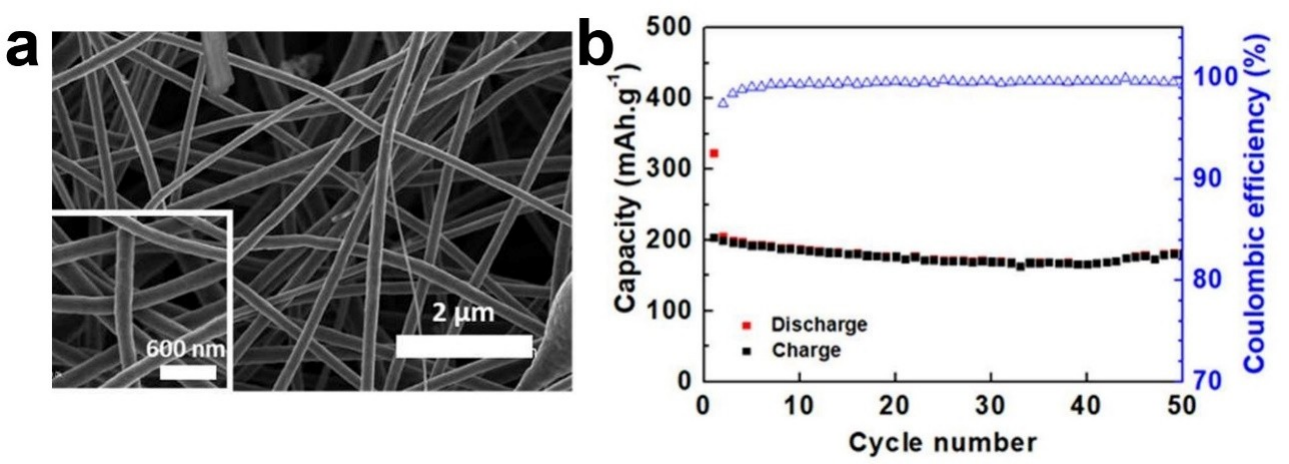
(a) High‐magnification field‐emission scanning electron microscopy (FESEM) image of CNFs. (b) Electrochemical performance of CNF at 25 mA g^−1^.^[16]^ Reproduced from Ref. [16] Copyright (2020), with permission from Elsevier.

In PIBs, due to the large ionic radius of K^+^, CNFs are difficult to exhibit excellent performance, and careful design is still needed to improve performances. For example, Touja *et al*. used PAN as raw material to prepare self‐supporting CNFs for PIBs anode by electrospinning technology, showing a general discharge capacity (200.0 mAh g^−1^ at 0.025 A g^−1^ after 50 cycles) (see Figure 1).^[16]^ Adams *et al*. obtained CNFs by electrospinning and subsequent carbonization.^[15]^ CNFs exhibit ordinary electrochemical performances (170 mAh g^−1^ at 0.279 A g^−1^ after 1900 cycles; 110 mAh g^−1^ at 2.79 A g^−1^).^[15]^

**Figure 2:**
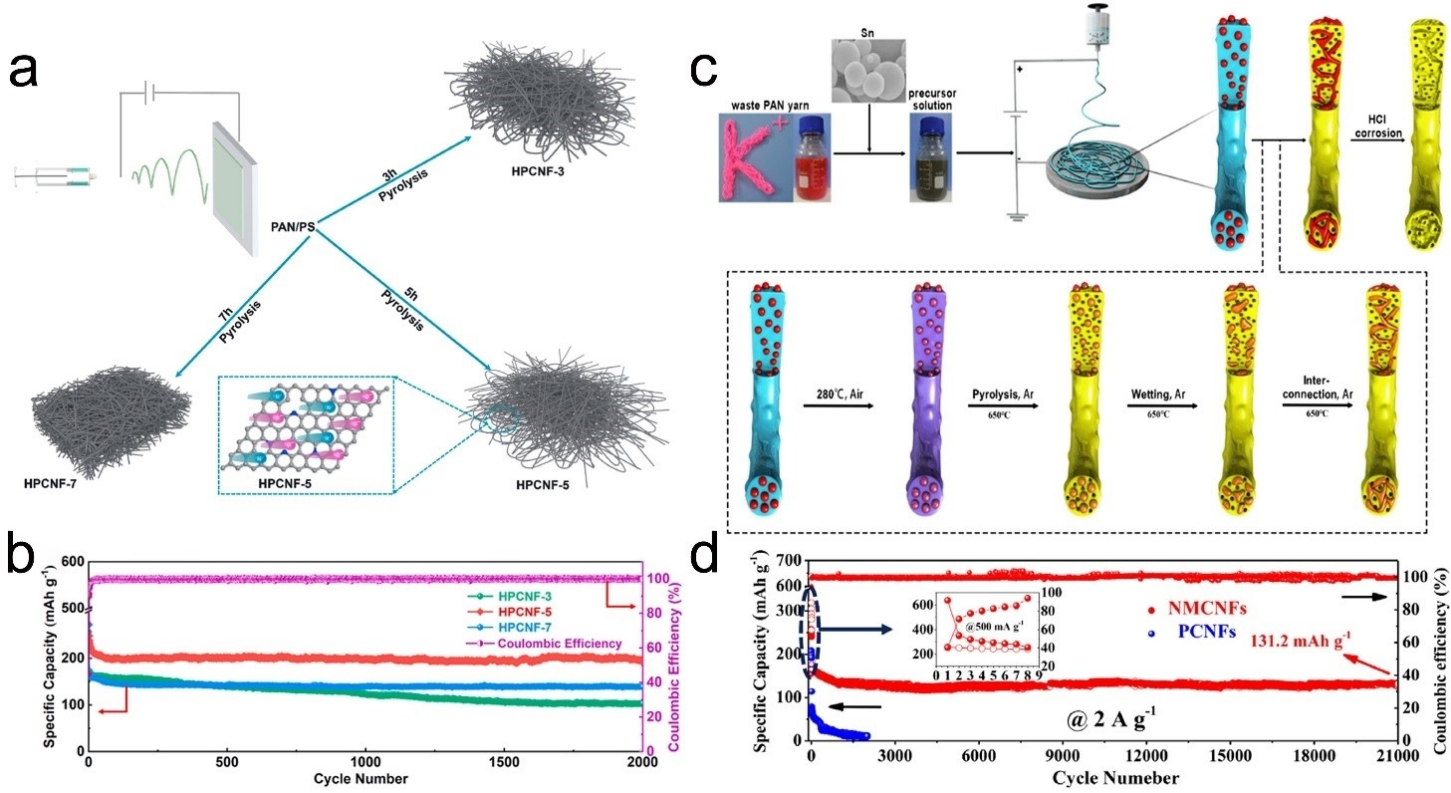
Schematic diagrams of the manufacturing process of (a) HPCNF and (c) NMCNFs, respectively.[^17^, ^20^] Ultra‐long cycle performances and coulombic efficiencies of (b) HPCNF and (d) NMCNFs, respectively.[^17^, ^20^] Reproduced from Ref. [17] Copyright (2022) and Ref. [20] Copyright (2020), respectively, with permission from Elsevier.

To enhance the electrochemical performances of CNFs, researchers have designed novel structures (such as manufacturing porous structures, and introducing other carbon materials, *etc*.), and introduced heteroatoms (such as S and N, *etc*.).[^17^, ^18^, ^19^] For example, Sun *et al*. successfully constructed in‐situ N‐doped hierarchical porous CNFs (HPCNF) using PAN and polystyrene (PS) as raw materials through electrospinning and pyrolysis processes (see Figure 2a).^[17]^ Among them, the decomposition of small molecular organic compounds forms a hierarchical pore structure and in‐situ N‐doping carbon. The HPCNF composite exhibits excellent rate performance (204.6 mAh g^−1^ at 2 A g^−1^), reversible capacity (282.9 mAh g^−1^ at 0.5 A g^−1^), and long cycle life [196.7 mAh g^−1^ at 2 A g^−1^ after 2000 cycles (see Figure 2b)], which can be attributed to defects and porous structure, increasing the contact area between the electrode and electrolyte, and improving K‐adsorption capacity. However, the HPCNF electrode has significant capacity loss during the initial cycling process, possibly due to the continuous formation of SEI layer due to the large number of pore structures. Liu *et al*. used the metal organic framework as a template to prepare self‐supporting porous, and N‐doped CNFs (NCNFs) by electrospinning and subsequent carbonization treatment.^[18]^ The NCNFs exhibit good reversible capacity (290 mAh g^−1^ at 0.1 A g^−1^), which can be attributed to the high surface area, tunable nitrogen species and abundant interconnected nanopores. Zhang *et al*. prepared N‐doped mesoporous CNFs (NMCNFs) using acrylic yarn and submicron tin by electrospinning and subsequent carbonization (see Figure 2c).^[20]^ The NMCNFs show a long‐term cycling stability [131.2 mAh g^−1^ at 0.5 A g^−1^ after 21000 cycles (see Figure 2d)]. This is mainly because its large interlayer spacing can adapt to large volume changes, as well as stable submicro‐sized and internal cross‐linking porous structure. However, the template method may lead to waste of raw material and increase preparation cost, and further optimization is needed in the future. Chen *et al*. synthesized a free‐standing flexible electrode material (CNT/SNCF) *via* electrospinning and sulfuration methods, realizing the high discharge capacity (212.5 mAh g^−1^ at 1 A g^−1^ after 1000 cycles) and cycling stability (100.1 mAh g^−1^ at 5 A g^−1^ after 5000 cycles) of K^+^ storage.^[19]^ The excellent storage capacity of CNT/SNCF can be attributed to the following: (i) the unique multi‐channel one‐dimensional structure can promote ion diffusion, and increase the conductivity and active sites of CNT/SNCF; (ii) S and N co‐doping improves electrochemical performance; and (iii) the increased interlayer spacing can mitigate volume changes during cycling.

Some synthesis methods and electrochemical properties of CNFs for PIBs are listed in Table 2. Obviously, introducing other carbon materials, pore forming agents, or heteroatoms (such as S and N, *etc*.) can effectively improve the cycling life and rate performance of electrode. These genius strategies can provide reference for the market application of CNFs anode for PIBs in the near future.

**Table 2:** Summary of the preparation methods and potassium storage performances of CNFs.

Material	Synthesis method (strategy)	Rate property (mAh g^−1^/A g^−1^)	Cycling stability (mAh g^−1^/A g^−1^/cycles)	Ref.
Self‐supporting CNFs	Electrospinning	150/1	200.0/0.025/50	[16]
CNFs	Electrospinning	~150/0.558 110/2.79	170/0.279/1900	[15]
HPCNF	Electrospinning (introducing pore forming agents)	234.5/1 204.6/2	238.6/1/200 196.7/2/2000	[17]
NCNFs	Electrospinning (introducing other carbon materials)	210/1 190/2 170/5	~250/0.1/100 150/5/1000	[18]
NMCNFs	Electrospinning and template methods (introducing pore forming agents)	230/1 161/5 96/15	351.1/0.2/500 131.2/0.5/21000	[20]
CNT/SNCF	Electrospinning and sulfuration methods (introducing other carbon materials, heteroatoms and pore forming agents)	190.6/1 108.7/5	355.9/0.1/100 212.5/1/1000 100.1/5/5000	[19]

### 2.2 Application of Metal/CNFs in PIBs

**Figure 3:**
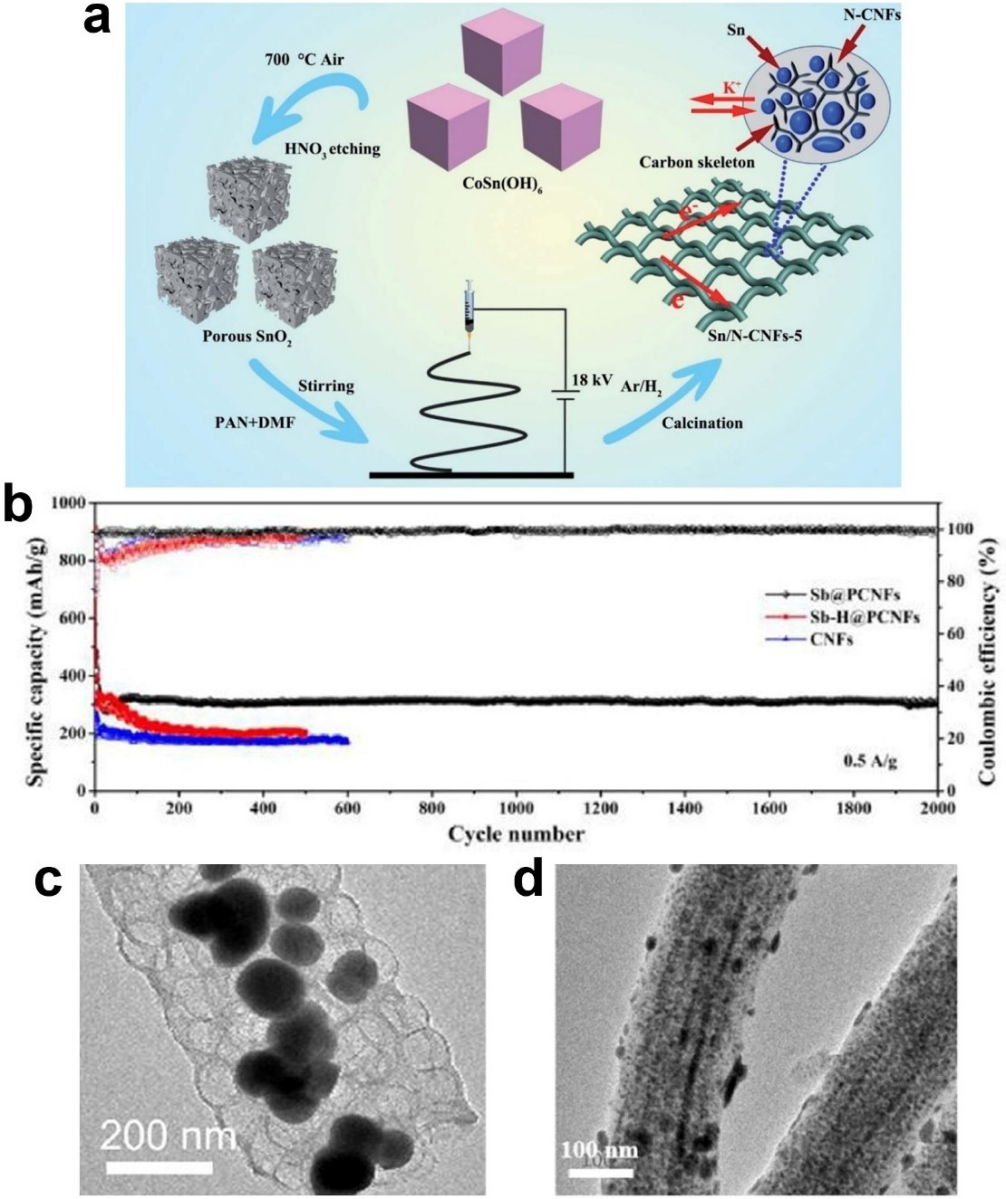
(a) Schematic diagram of the manufacturing process of Sn/N‐CNFs.^[5]^ Reproduced from Ref. [5] Copyright (2021), with permission from Royal Society of Chemistry. (b) Cycling performance of Sb@PCNFs electrode at 500 mA/g.^[19]^ Reproduced from Ref. [19] Copyright (2021), with permission from American Chemical Society. (c) transmission electron microscopy (TEM) image of BiSb@C composite.^[22]^ Reproduced from Ref. [22] Copyright (2022), with permission from American Chemical Society. (d) TEM image of the Bi/PCNFs.^[23]^ Reproduced from Ref. [23] Copyright (2022), with permission from American Chemical Society.

Recently, high‐capacity metal anode materials based on alloying reactions have been receiving increasing attention due to low charge/discharge potentials, low cost, and high theoretical capacity. Such materials mainly include metals, such as Sb, Sn and Bi, and their alloys, which have been extensively studied in LIBs and sodium‐ion batteries (SIBs).^[21]^ However, metal materials typically face significant volume expansion (>300 %) during cycling, which may lead to rapid capacity decay.^[5]^ At present, there are two main strategies to solve this problem: the combination of metal materials with carbon materials, such as graphene, CNFs, and porous carbon, *etc*.; and the design of novel structures, such as reducing size, and introducing porous structures, *etc*.[^5^, ^21^] For example, Li *et al*. used porous SnO_2_ and PAN as precursors to prepare porous Sn/N‐doped CNFs frameworks (Sn/N‐CNFs) by electrospinning and carbonization methods (see Figure 3a).^[5]^ The Sn/N‐CNFs exhibits the outstanding electrochemical properties (198.0 mAh g^−1^ at 1 A g^−1^ after 3000 cycles, the corresponding capacity retention rate is as high as 88.4 %; 316.1 mAh g^−1^ at 0.1 A g^−1^ after 100 cycles; 168.7 mAh g^−1^ at 2 A g^−1^) as the anode material for PIBs. The excellent storage capacity of Sn/N‐CNFs may be due to the following reasons: (i) porous Sn nanospheres provide sufficient space to accommodate volume changes during the alloying process, and improve potassium storage performances; (ii) the amorphous carbon skeleton inhibits the aggregation of metal nanoparticles; and (iii) the N‐doped CNFs can ensure the stability of the structure. However, the first coulombic efficiency of Sn/N‐CNFs in this work is relatively low, not exceeding 80 %. In order to improve this issue, the authors propose a strategy to optimize the electron/ion transport capacity, which involves increasing the content of Sn and increasing the carbonization temperature to enhance the conductivity of CNFs. Chen *et al*. used polyoxyethylene‐polyoxypropylene‐polyoxyethylene triblock copolymer (P123), SbCl_3_ and PAN as precursors to prepare Sb@porous N‐doped CNFs (Sb@PCNFs) by electrospinning and carbonization methods.^[21]^ Among them, the addition of P123 can be used to manufacture pore structures, which alleviates the volume changes during cycling and improves the transport capacity of electrons/ions. As an anode electrode material for PIBs, Sb@PCNFs hybrid exhibits superior cycling performance (314 mAh g^−1^ at 0.5 A g^−1^ after 20000 cycles) (see Figure 3b). Considering the high capacities and rate performances of Sb and Bi, respectively. Li *et al*. used SbCl_3_, Bi(NO_3_)_3_ ⋅ 5H_2_O and PAN as raw materials to prepare BiSb nanoparticles/CNFs composites (BiSb@C) as PIBs anode by electrospinning and carbonization methods, showing good cycle stability and capacity (204.8 mAh g^−1^ at 0.1 A g^−1^ after 500 cycles, the corresponding capacity retention rate is as high as 82.8 %).^[22]^ The excellent electrochemical performances for BiSb@C are due to the carbon coating can mitigate the volume change of BiSb, as well as the CNFs could improve the conductivity and structural stability (see Figure 3c). Ouyang *et al*. used Bi(NO_3_)_3_, PAN, and PVP as raw materials to prepare Bi nanoparticles/porous CNFs composites (Bi/PCNFs) as PIBs anode by electrospinning and carbonization methods, showing excellent performances (171 mAh g^−1^ at 1 A g^−1^ after 1000 cycles; 163.3 mAh g^−1^ at 5 A g^−1^).^[23]^ The good cycle life of Bi/PCNFs can be attributed to the following: (i) carbon coating, relieving the mechanical strain (see Figure 3d); (ii) the unique vessel like pores, enhancing the contact area between the electrode and electrolytes; and (iii) the optimal ratio between the Bi and the carbon, improving capacity and cycle life. Han *et al*. prepared N‐doped CoSb@carbon nanofiber (CoSb@C) composite materials by electrospinning and carbonization with Co(CH_3_COO)_2_ ⋅ 4H_2_O and SbCl_3_ as the raw materials of CoSb alloy, and PAN as the carbon source.^[24]^ Among them, N and Co can not only optimize the electronic configuration but also alleviate the volume expansion during cycling. As a PIBs anode material, CoSb@C shows excellent performance (413 mAh g^−1^ at 1 A g^−1^ after 1000 cycles, the corresponding capacity retention rate is as high as 82.8 %).^[24]^

Some synthesis methods and electrochemical properties of metal/CNFs for PIBs are listed in Table 3. In conclusion, the combination of metal materials and CNFs can effectively accommodate the volume change of metal during cycling and improve the long cycle performance of the electrode. However, the above composites usually face the problem of low the first coulombic efficiency and low capacity. Reducing the use of carbon materials and optimizing the experimental design are the focus of the next research step.

**Table 3:** Summary of the preparation methods and potassium storage performances of metal/CNFs.

Material	Synthesis method	Rate property (mAh g^−1^/A g^−1^)	Cycling stability (mAh g^−1^/A g^−1^/cycles)	Ref.
Sb@PCNFs	Electrospinning and carbonization methods	~110/5	314/0.5/2000	[21]
Sn/N‐CNFs	Electrospinning and carbonization methods	168.7/2	316.1/0.1/100 198/1/3000	[5]
BiSb@C	Electrospinning and carbonization methods	70/2	204.8/0.1/500	[22]
Bi/PCNFs	Electrospinning and carbonization methods	163.3/5	171/1/1000	[23]
CoSb@C	Electrospinning and carbonization methods	161/5 96/15	413/1/1000	[24]

### 2.3 Application of Chalcogenide/CNFs in PIBs

**Figure 4:**
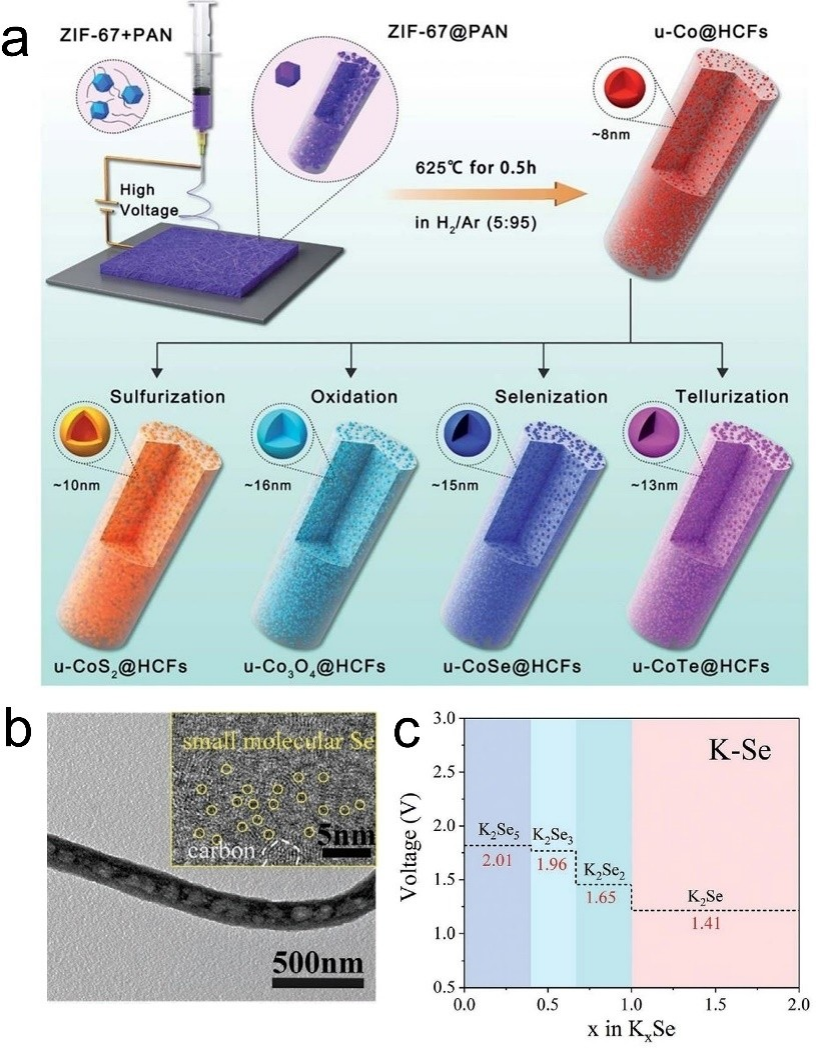
(a) Schematic diagram of the manufacturing process of ultrasmall CoM_x_ (M=S, O, Se and Te)@HCFs composites.^[8]^ Reproduced from Ref. [8] Copyright (2021), with permission from Royal Society of Chemistry. (b) TEM image with the high‐resolution TEM image (inset) of Se@NPCFs.^[35]^ (c) Computed voltage curve of K_x_Se systems.^[35]^ Reproduced from Ref. [35] Copyright (2020), with permission from Wiley‐VCH.

Recently, chalcogenide compounds, such as SnS_2_, Sb_2_Se_3_, CoSe, FeSe and VS_4_, have been widely used in the field of rechargeable batteries due to their high theoretical capacity.[^25^, ^26^, ^27^, ^28^] However, chalcogenide compounds are generally facing many challenges including large volume expansion rate and poor conductivity during cycling, which seriously affect the cycle performance, rate performance and discharge capacity of batteries, especially in PIBs. At present, researchers mainly use the following methods to solve this problem: carbon coating, such as CNFs, and graphene, *etc*;^[29]^ reasonable structural design, such as reducing the size of chalcogenide, introducing porous structure and constructing heterojunction structure, *etc*;[^30^, ^31^, ^32^] introducing heteroatoms, such as metal, and O, *etc*.[^26^, ^33^, ^34^] Among them, CNFs, as an important substrate material, often have positive effects when combined with chalcogenide compounds. For example, Luo *et al*. used acetylacetone iron, graphite oxide and PAN as raw materials to prepare FeSe@Graphene@CNFs (FeSe@G@CNFs) by electrospinning and selenidation.^[25]^ FeSe@G@CNFs hybrid exhibit excellent electrochemical performances (200 mAh g^−1^ at 2 A g^−1^ after 1700 cycles, the corresponding capacity retention rate is as high as 80.9 %; 409 mAh g^−1^ at 0.2 A g^−1^ after 400 cycles; 245 mAh g^−1^ at 5 A g^−1^). The good electrochemical performance can be attributed to the fact that with the help of graphene, more FeSe nanoparticles are encapsulated in CNFs, which can alleviate the volume change of FeSe during cycling and shorten the diffusion distance of ions. However, these experimental steps for this work are complicated, and the introduction of graphite oxide also increases the cost of raw materials. Wang *et al*. synthesized a few layers of Sn‐based sulfide‐N/P co‐doped CNFs hybrid (SnS_x_‐N/P‐CNFs) with chlorella as the precursor through electrospinning technology and vulcanization process.^[26]^ The SnS_x_‐N/P‐CNFs hybrid shows the outstanding potassium storage properties (468 mAh g^−1^ at 0.1 A g^−1^ after 100 cycles; 170 mAh g^−1^ at 5 A g^−1^ after 10000 cycles). It is worth mentioning that the use of biomass algae as a N/P sources can improve the conductivity of the electrode material, increasing the electrochemical performances of the electrode.^[26]^ Lai *et al*. used ZIF‐67 and PAN as raw materials to prepare ultrasmall CoM_x_ (M=S, O, Se and Te)@hierarchical porous CNFs (HCFs) composites by electrospinning, carbonization and subsequent sulfuration/oxidation/selenidation/tellurization methods (see Figure 4a), indicating that this method has good flexibility.^[8]^ Among them, ultrasmall CoS_2_ (u‐CoS_2_)@HCFs exhibits excellent long cycle performance (268 mAh g^−1^ at 0.5 A g^−1^ after 1000 cycles). The outstanding potassium storage properties can be ascribed to the synergistic effects of the well‐organized ultrasmall CoS_2_ nanoparticles with tight coupling to the hierarchically porous CNFs. Inspired by the favorable structure of pea‐pod, Xu *et al*. used SiO_2_ as a template to prepare pea‐pod‐like porous CNFs through electrospinning, carbonization and activation methods.^[35]^ Subsequently, by a melting liquefaction strategy, Se is infiltrated into the prepared porous CNFs to obtain self‐supporting Se@peapod‐like N‐doped CNFs (Se@NPCFs) composite materials (see Figure 4b). The Se@NPCFs hybrid has an excellent long cycle life (367 mAh g^−1^ at 0.5 A g^−1^ after 1670 cycles).^[35]^ The good cycle life of Bi/PCNFs can be attributed to the following: (i) 1D peapod‐like structure, facilitating the electrolyte infiltration and K^+^ diffusion; (ii) the abundant micro/mesopores, increasing mass loading of Se, shortening the ion diffusion distance and buffering the volume expansion; and (iii) N‐doping carbon, enhancing the chemical affinity between the carbon matrix and discharge products. Besides, this work further analyzed the reaction mechanism of Se during the discharge process through density functional theory (DFT). The theoretical potential corresponding to the K_2_Se and K_2_Se_2_ (1.41 and 1.65 V, respectively) formed during the discharge process is shown in Figure 4c, which is higher than the experimental value (1.20 and 1.45, respectively). The possible reason is the overpotential caused by the electrode and electrolyte.

Some synthesis techniques and electrochemical characteristics of chalcogenide/CNFs for PIBs are listed in Table 4. The combination of chalcogenide and CNFs is an effective method to improve the long cycle life of chalcogenide. However, the experimental methods involved in the combination of these two are mostly complex and require careful design to reduce the number of steps for future market applications.

**Table 4:** Summary of the preparation methods and potassium storage performances of chalcogenide/CNFs.

Material	Synthesis method	Rate property (mAh g^−1^/A g^−1^)	Cycling stability (mAh g^−1^/A g^−1^/cycles)	Ref.
FeSe@G@CNFs	Electrospinning and selenidation	245/5	409/0.2/400 200/2/1700	[25]
SnS_x_‐N/P‐CNFs	Electrospinning technology and vulcanization process	101/10	468/0.1/100 170/5/10000	[26]
Se@NPCFs	Electrospinning, carbonization, activation methods, and melting liquefaction strategy	209/2	635/0.05/50 367/0.5/1670	[35]
u‐CoS_2_@HCFs	Electrospinning, carbonization and sulfuration methods	~220/5	268/0.1/100 268/0.5/1000	[8]

### 2.4 Application of Phosphide/CNFs in PIBs

**Figure 5:**
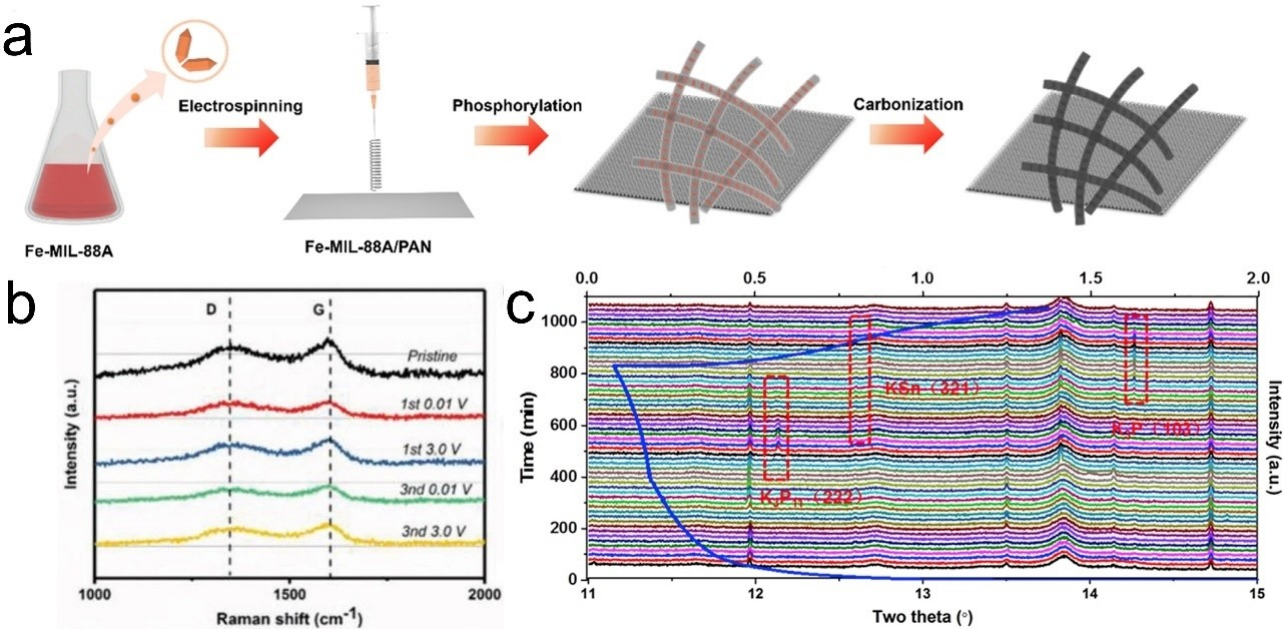
(a) Schematic diagram of the manufacturing process of Fe_2_P‐CNFs.^[38]^ Reproduced from Ref. [38] Copyright (2022), with permission from Elsevier. (b) Raman spectra of pristine MoP@NPCNFs anode and the anodes after the 1^st^ discharge, 1^st^ charge, 3^rd^ discharge and 3^rd^ charge.^[36]^ Reproduced from Ref. [36] Copyright (2020), with permission from Wiley‐VCH. (c) In operando synchrotron XRD patterns of Sn_4_P_3_ as anode for PIBs.^[39]^ Reproduced from Ref. [39] Copyright (2018), with permission from Elsevier.

Phosphides attract the interest of researchers due to its high theoretical capacity (for example, the theoretical capacity of boron phosphide is 570 mAh g^−1^) and low voltage platform (~0.4 V). However, the large volume expansion, poor conductivity (for example, ~10^−14^ S cm^−1^ for red phosphorus) and sluggish kinetics during charging/discharging prevent its practical application.[^36^, ^37^] As a stable carbon substrate, CNFs have been used to improve the electrochemical performance of phosphide. For example, Sun *et al*. used MIL‐88A (Fe) and PAN as raw materials to obtain self‐supporting Fe_2_P nanoparticle doped CNFs (Fe_2_P‐CNFs) composites through electrospinning, phosphorylation and carbonization (see Figure 5a).^[38]^ The Fe_2_P‐CNFs hybrid has good reversible capacity (379.2 mAh g^−1^), rate performance (211.8 mAh g^−1^ at 2 A g^−1^), and long cycle life (179.6 mAh g^−1^ at 2 A g^−1^ after 2000 cycles). The excellent electrochemical performances of Fe_2_P‐CNFs can be attributed to the following: (i) the incorporation of Fe_2_P nanoparticles, improving intrinsic electronic conductivity and introducing a large number of defects; and (ii) amorphous CNFs, providing abundant active sites and enlarging interlayer spacing. Note that the proportion of Fe_2_P in Fe_2_P‐CNFs calculated is 8.8 % based on thermogravimetric analysis results. Such a low load may affect the volume energy density of the battery. Therefore, it is necessary to develop phosphide electrode materials with high load. Yi *et al*. used ZIF‐8 and PAN as raw materials to prepare self‐supporting MoP@N, P co‐doped CNFs (MoP@NPCNFs) composites by electrospinning, carbonization and phosphating, exhibiting good electrochemical performances (220 mAh g^−1^ at 2 A g^−1^ after 100 cycles; 320 mAh g^−1^ at 0.1 A g^−1^) as PIBs anode electrode.^[36]^ These remarkable performances originate from the following merits: (i) the NPCNFs can alleviate the P pulverization during cycling and serve as the mechanical supporting and electronic conductive network, improving the stable cycling and excellent rate capability; and (ii) the N, P co‐doping could enhance the wettability of the electrode, improving the efficient mass transfer during cycling. To investigate the reaction mechanism of MoP@NPCNFs, Raman spectra was applied to follow structural evolution after different cycling (see Figure 5b). From Figure 5b, it can be observed that the characteristic D (~1350 cm^−1^) and G (~1585 cm^−1^) bands of the pristine MoP@NPCNFs electrode. The intensity ratio of *I*_D_/*I*_G_ decreased after the 1^st^ discharge, indicating more defects and disorders in the carbon structure, which may be caused by K^+^ insertion. After fully charged, the value of *I*_D_/*I*_G_ is restored again. The same situation is also observed for the 3^rd^ discharge and charge, indicating the stability of the electrode structure. Note that the first coulombic efficiency of MoP@NPCNFs is inefficient (not more than 40 %), which needs to be further improved. Zhang *et al*. prepared Sn_4_P_3_@CNFs as PIBs anode using Sn, P and PAN as raw materials by ball milling and electrospinning.^[39]^ In this work, the authors found that potassium bis(fluorosulfonyl)imide can effectively inhibit the growth of potassium dendrites, reduce the impact of polarization, ensure the stable SEI layer, and allow for long‐term stable operation of the electrode. Thus, the composite material exhibits good cyclic stability (160.7 mAh g^−1^ at 0.5 A g^−1^ after 1000 cycles). In addition, the authors also found that the potassiation process could be described by the following equations based on synchrotron X‐ray diffraction (XRD) results (see Figure 5c):
(1)

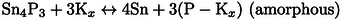



(2)

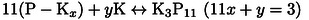



(3)

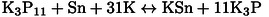




Some preparation methods and electrochemical performances of phosphide/CNFs for PIBs are listed in Table 5. The use of CNFs to encapsulate phosphides seems to effectively improve the conductivity of electrodes and alleviate the volume expansion of nanoparticles.

**Table 5:** Summary of the preparation methods and potassium storage performances of phosphide/CNFs.

Material	Synthesis method	Rate property (mAh g^−1^/A g^−1^)	Cycling stability (mAh g^−1^/A g^−1^/cycles)	Ref.
Fe_2_P‐CNFs	Electrospinning, phosphorylation and carbonization	211.8/2	179.6/2/2000	[38]
MoP@NPCNFs	Electrospinning, carbonization and phosphating	320/0.1	220/2/100	[36]
Sn_4_P_3_@CNFs	Ball milling and electrospinning		160.7/0.5/1000	[39]

### 2.5 Application of Other Materials/CNFs in PIBs

**Figure 6:**
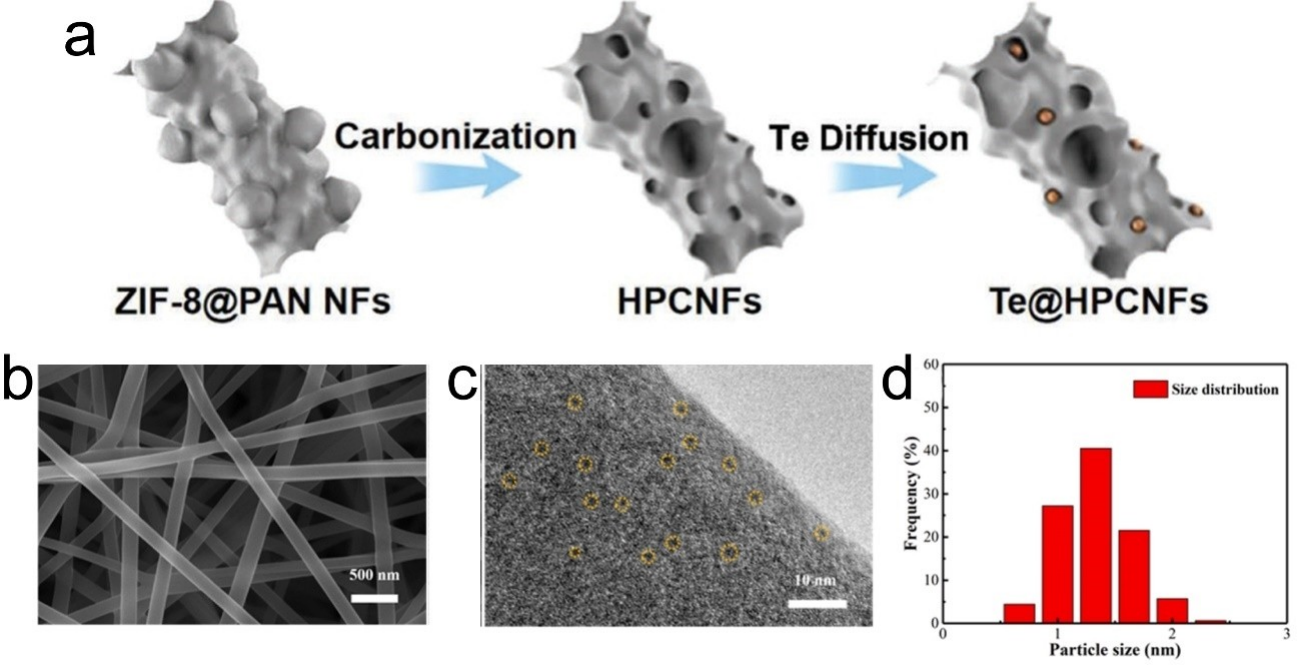
(a) Schematic diagram of the manufacturing process of Te@HPCNFs.^[42]^ Reproduced from Ref. [42] Copyright (2022), with permission from Wiley‐VCH. (b) FESEM image, (c) TEM image and (d) corresponding particle size distribution image of Mo_2_C/NCNFs, respectively.^[43]^ Reproduced from Ref. [43] Copyright (2021), with permission from Elsevier.

In addition to the materials mentioned above, there are other materials combined with CNFs, such as quasi‐metals (Te, *etc*.), and carbides (Mo_2_C, *etc*.). For quasi‐metallic Te, it has been extensively studied in alkali metal‐ion batteries due to its high density (6.25 g cm^−3^), attractive theoretical capacity (419 mAh g^−1^), and excellent electron conductivity (2102 S m^−1^).[^40^, ^41^, ^42^] However, the alkali metal‐Te cells are affected by the unfavorable shuttling effect of the polytetrafluoroethylene intermediate, leading to the loss of active materials and severely weakening the electrochemical stability of the two electrodes. In addition, Te material undergoes huge volume expansion (398 % for K_2_Te) during charging and discharging processes, resulting in rapid capacity decay.[^40^, ^42^] In order to solve the above problems, an effective approach is to confine Te into porous carbon. For example, Yu *et al*. used zeolite imidazolate backbone material (ZIF‐8) and PAN as raw materials, and prepared Te@hierarchical porous CNFs (Te@HPCNFs) composite material as PIBs anode electrode by electrospinning method and melt‐diffusion method (see Figure 6a), showing excellent electrochemical properties (231.7 mAh g^−1^ at 7C after 4500 cycles; 207.1 mAh g^−1^ at 14C).^[42]^ The unprecedented cycle life of Te@HPCNFs can be attributed to the space confinement of HPCNFs, which accommodates the volume expansion of amorphous Te during cycling. Besides, Te@HPCNFs hybrid also has a superior capacity in K‐ion full cell (170.3 mAh g^−1^ at 0.1 A g^−1^ after 100 cycles). In this work, the authors comprehensively examined the electrochemical performances of Te@HPCNFs, which help readers understand the performances of Te‐based materials in PIBs.

Other uncommon electrode materials have also been reported to be combined with CNFs for PIBs, such as Mo_2_C. For example, Min *et al*. used (NH_4_)_6_Mo_7_O_24_ ⋅ 4H_2_O and PAN as raw materials to prepare self‐supporting Mo_2_C/N‐doped CNFs (Mo_2_C/NCNFs) electrode material as PIBs anode through electrospinning and carbonization methods (see Figure 6b).^[43]^ The electrode material exhibits general properties (88 mAh g^−1^ at 1 A g^−1^ after 1000 cycles; 211 mAh g^−1^ at 0.1 A g^−1^). In this work, the average diameter of Mo_2_C quantum dots is 1–2 nm (see Figure 6c and 6d), which can shorten the electron/ion migration distance and improve charge transfer kinetics. However, the loading amount of Mo_2_C in this material is found to be 17 wt %, resulting in lower levels of performance.

In conclusion, when CNFs are compounded with quasi‐metals (Te, *etc*.), and carbides (Mo_2_C, *etc*.), *etc*, these hybrids can be guaranteed to have good cycle stability. However, the loading capacity of these materials in CNFs is not high, which affects the volume energy density of the entire battery. Therefore, the next focus can be on increasing the proportion of active substances in hybrid. In addition, how to reduce the specific surface area of the electrode material and improve the first coulomb efficiency of the electrode should also focus.

## 3 Conclusions

Currently, LIBs have been widely used, and SIBs batteries are also in the preparation stage for marketization. It is believed that PIBs will also be applied in the near future. In this paper, the research progress and key problems of CNFs‐based materials (including CNFs, metal/CNFs composites, chalcogenide/CNFs composites, phosphide/CNFs composites and other CNFs‐based materials) in PIBs are mainly discussed on the basis of predecessors. Among them, although CNFs‐based materials have received widespread attention from researchers due to their excellent structural stability, conductivity and long cycle life. However, CNFs‐based materials still face many challenges such as low capacity, low first cycle coulombic efficiency, unclear reaction mechanism, complex synthesis method and high cost. Future research may be conducted as follows:

- It is evident that introducing heteroatoms, pore structures, and other components, *etc*, can significantly improve the electrochemical performance of CNFs. However, those unique structures can only be prepared in the laboratory. How to simplify production processes and reduce production costs to meet the needs of industrial production is the focus of the next step of research.
- Advanced characterization techniques (such as in‐situ XRD, TEM, Raman, *etc*.) and theoretical methods (such as, DFT calculation, *etc*.) are needed to deeply analyze the structural characteristics of CNFs‐based materials, guide their composition and structural design, and analyze the structural changes of electrode materials during cycling.
- There is relatively little research on the coordination of CNFs‐based materials with other battery components (such as positive electrodes, separators, electrolytes, *etc*.). More research should focus on these directions to improve the overall performances of battery.

In summer, with the continuous iteration and upgrading of technology and the increasing market popularity, it is believed that CNFs‐based materials can play a huge role in PIBs.

## Conflict of interests

The authors declare no conflict of interest.

4

## Biographical Information

*Chao Li received his Ph.D. from Jilin University in 2021. He is currently working as a postdoctoral fellow at Research Institute of Sinopec Maoming Company. His research interests focus on energy conversion and energy storage materials*.

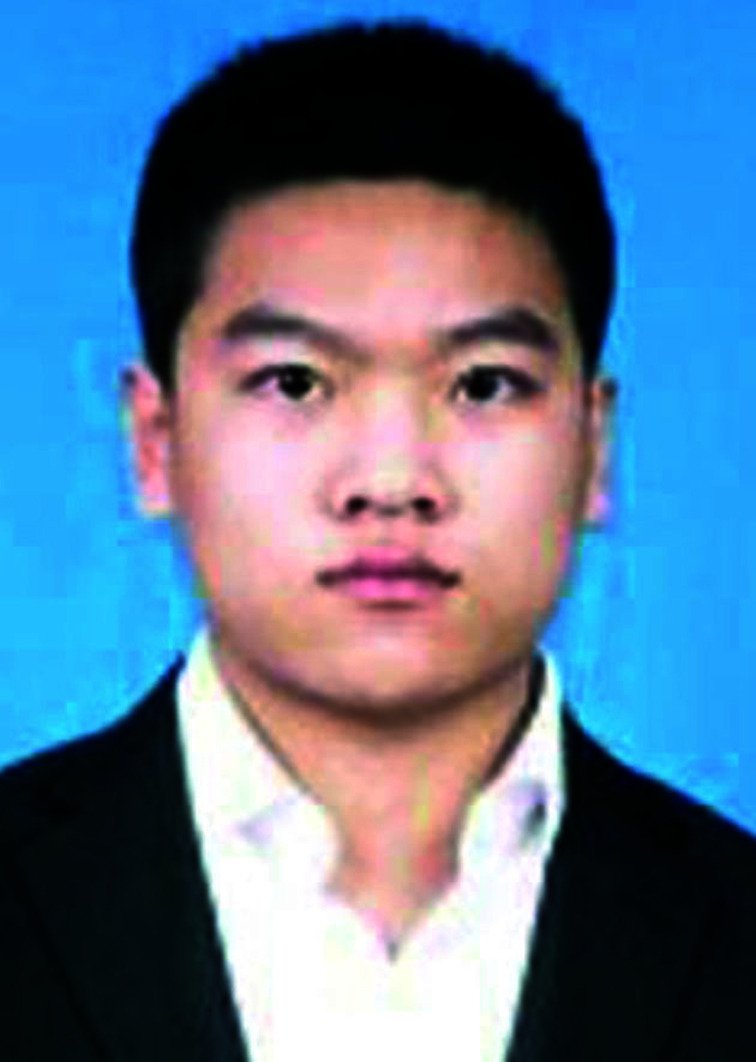

## Biographical Information

*Wen‐jun Jiang obtained a Master degree from Hunan University of Science and Technology in 2008. He is currently working as a senior engineer at Research Institute of Sinopec Maoming Company. His research interests focus on development and utilization of organic fine chemicals*.

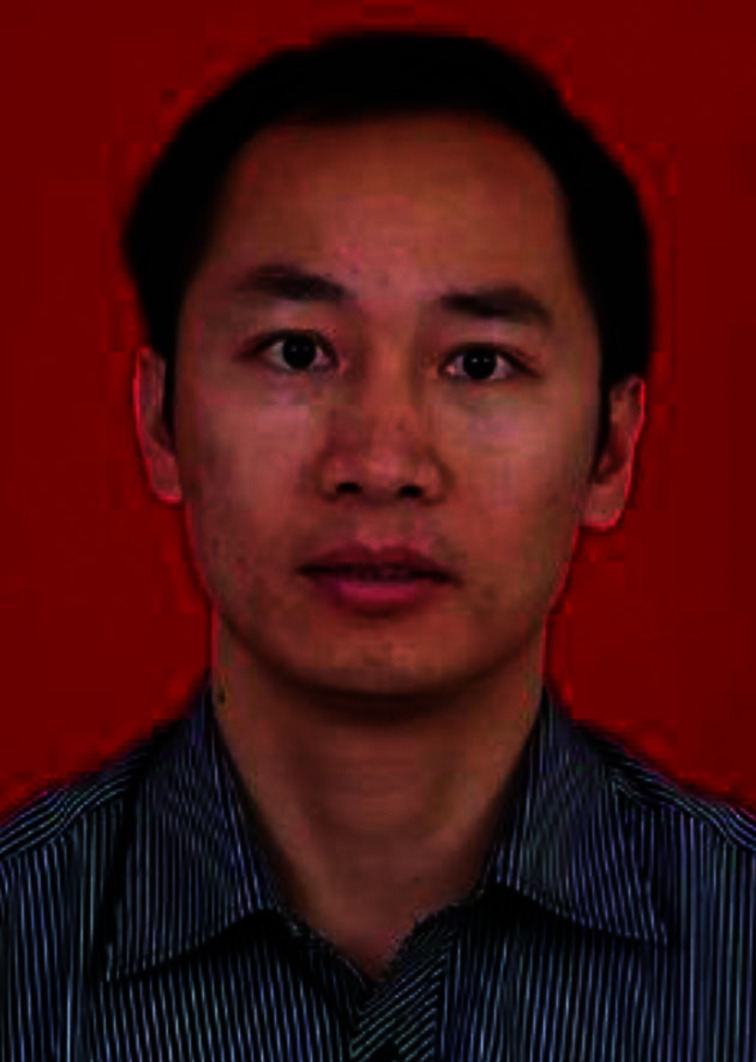

## Biographical Information

*Zhen‐yu Liu earned his Ph.D. from Beijing University of Chemical Technology in 2013. He is currently working as a senior engineer at Research Institute of Sinopec Maoming Company. His research interests focus on organic fine chemicals, olefin polymerization catalysts, and new product development*.

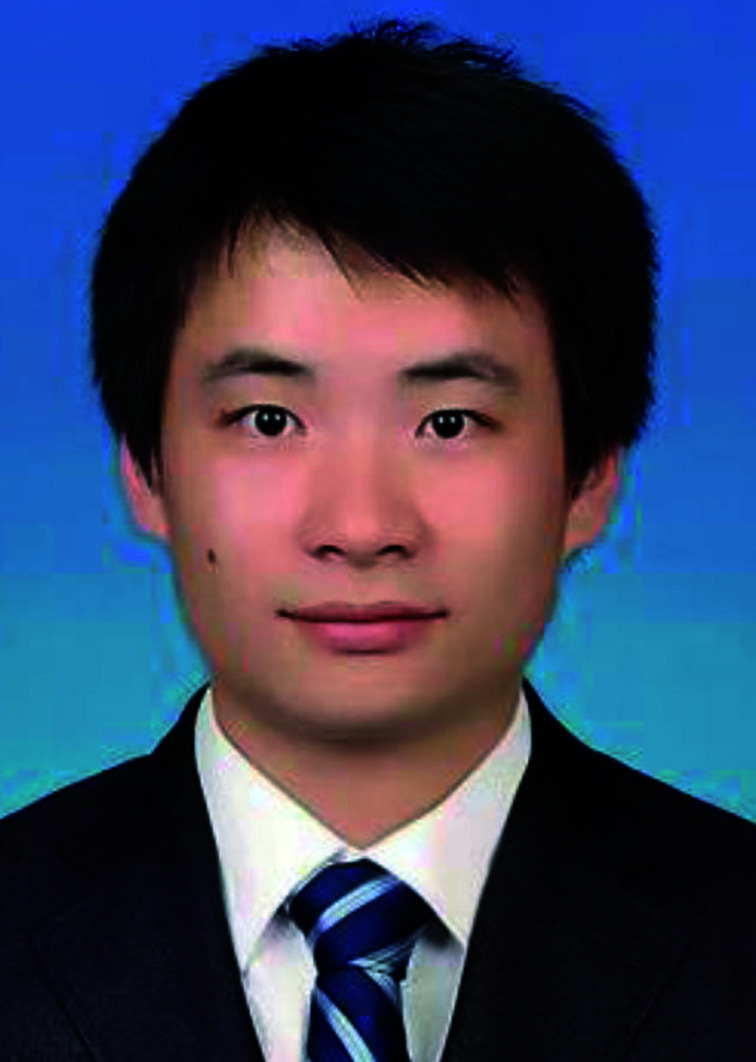

## Data Availability

The data that support the findings of this study are available from the corresponding author upon reasonable request.
